# Detecting microstructural properties of white matter based on compartmentalization of magnetic susceptibility

**DOI:** 10.1016/j.neuroimage.2012.12.032

**Published:** 2013-04-15

**Authors:** Way Cherng Chen, Sean Foxley, Karla L. Miller

**Affiliations:** a Centre for Functional MRI of the Brain, University of Oxford, Oxford, UK; b Singapore Bioimaging Consortium, A*STAR, Singapore

**Keywords:** GRE phase images, *R*_2_*, Magnetic susceptibility

## Abstract

The microscopic structure of neuronal tissue is crucial to brain function, with axon diameter, axonal density and myelination directly influencing signal conduction in the white matter. There is increasing evidence that these microstructural properties alter signal in magnetic resonance imaging (MRI) driven by magnetic susceptibility of different compartments (e.g., myelin sheaths and iron-laden cells). To explain these observations, we have developed a multi-compartmental geometric model of whitematter microstructure. Using a single set of literature parameters, this forward model predicts experimentally observed orientation dependence and temporal evolution of the MRI signal. Where previous models have aimed to explain only the orientation dependence of signal phase, the proposed approach encapsulates the full repertoire of signal behavior. The frequency distribution underlying signal behavior is predicted to be a rich source of microstructural information with relevance to neuronal pathology.

## Highlights

► A multi-compartmental geometric model of white matter microstructure is introduced ► Successfully predicted experimentally-observed orientation dependence of MRI signal ► Successfully predicted experimentally-observed temporal evolution of MRI signal ► Proposed approach encapsulates the full repertoire of signal behavior

## Introduction

White matter (WM) in the brain is composed of a highly complex cellular microstructure, consisting primarily of myelinated axons, glia, vasculature and interstitial space. This microarchitecture is fundamental to brain function, varies across different brain regions and is compromised in a range of neurodegenerative disorders. In general, the microstructural compartments have unique magnetic susceptibilities driven by their chemical compositions and molecular arrangements. In the presence of an external magnetic field, differences in magnetic susceptibilities between adjacent compartments generate local magnetic field perturbations, and hence a range of magnetic resonance frequencies. The precise field perturbation depends on the geometry of the compartments, their spatial arrangement and direction of the main magnetic field, *B*_0_. This raises the intriguing possibility that this microenvironment might be reflected in MR signal changes driven by the magnetic field distribution within an imaging voxel. There is a long-standing literature on signal changes due to partially-oxygenated blood vessels, but brain parenchymal microstructure has received less attention.

Gradient echo (GRE) MR techniques that are sensitive to magnetic susceptibility effects, such as *R*_2_* mapping and phase imaging, have been proposed to probe various aspects of WM microstructure (Duyn et al., 2007; Fukunaga et al., 2010; He and Yablonskiy, 2009; Lee et al., 2010a), with recent interest due to the high contrast afforded by ultra-high field strength scanners (≥ 7 Tesla). Correlation of *R*_2_* and GRE phase images with non-heme iron (primarily the iron storage protein, ferritin) have been reported in the gray matter (Fukunaga et al., 2010; Gelman et al., 1999; Haacke et al., 2005; Ogg et al., 1999; Schipper, 2012; Yao et al., 2009); however, the relationship between WM regions is less studied. The degree of myelination has been shown to significantly affect both *R*_2_* and phase in WM (Lee et al., 2012; Liu et al., 2011; Lodygensky et al., 2012). Recent studies have reported modulation of *R*_2_* and phase with orientation of the WM fiber to *B*_0_ (Bender and Klose, 2010; Cherubini et al., 2009; Denk et al., 2011; He and Yablonskiy, 2009; Lee et al., 2010a, 2011; Liu, 2010; Marques et al., 2009; Sati et al., 2012), which strongly implicates magnetic susceptibility as the origin of this effect.

Several mechanisms have been proposed to explain the observed signal properties, including magnetic susceptibility anisotropy (Lee et al., 2010a; Liu, 2010) and the presence of cylindrical susceptibility-shifted inclusions (He and Yablonskiy, 2009). These previous works have proposed models to explain orientation dependence of the signal phase and *R*_2_* decay, which are driven by the first and second moments (mean and variance) of the frequency distribution, respectively. These models have attributed effects such as orientation dependence to the microenvironment, but without explicitly modeling the microstructure. For example, signal phase has been modeled as a mixture of susceptibilities with either intrinsic (Lee et al., 2010a) or apparent (He and Yablonskiy, 2009) orientation dependence. While providing a closed-form expression for the orientation dependence of signal phase, these models are designed to explain a limited range of signal behaviors.

In the present work, we introduce an explicit multi-compartmental geometric model of the WM that encapsulates magnetic properties to investigate the contribution of the tissue microarchitecture in generating signal properties. The power of this approach is that it predicts the entire frequency distribution contained within a voxel, not just the low-order moments, capturing the full richness of information in this distribution. This work is closely related to susceptibility models of the vasculature, but with a crucial difference: low blood volume results in distributions that are well-characterized by the field patterns surrounding an isolated blood vessel (Yablonskiy and Haacke, 1994), whereas the field perturbations surrounding densely-packed WM axons interact such that the field distribution depends on the packing geometry. Modeling this geometry explicitly provides a simple but powerful approach that is able to describe both low- and high-order moments (Miller et al., 2010; van Gelderen et al., 2012). Specifically, we demonstrate that this model predicts the experimentally-observed dependence of signal phase and *R*_2_* (low-order moments) on the orientation of WM fiber to *B*_0_. High-order moments are expected to encapsulate interesting aspects of compartmentalization to which lower-order moments are less sensitive. We demonstrate that the model agrees with measurements of the deviation of signal behavior from low-order moment approximations, as indicated by non-linear phase and non-mono-exponential magnitude time courses (van Gelderen et al., 2012). We then use the model to predict changes due to two sources of susceptibility contrast in WM: demyelination and iron concentration. These properties are physiologically relevant to WM function, plasticity and disease, making GRE signal measures potential biomarkers for myelin and iron.

## Materials and methods

### Geometric model

**Fig. 1:**
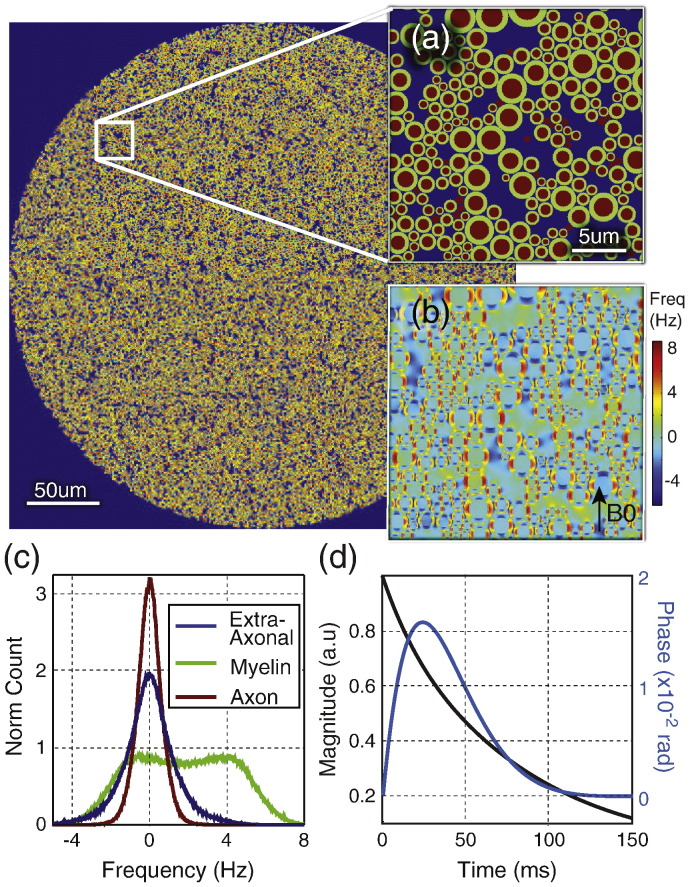
2D geometric model of white matter (WM). (a) Approximately 50000 WM fibers were modeled in a circular bundle with a close random packing. White box shows a zoomed in illustration of the WM microstructure. Dark blue = extra-axonal space, green = myelin, red = axon. (b) The frequency map generated in presence of an external magnetic field ***B*_0_**. (c) Frequency histogram. (e) The resultant magnitude decay and phase evolution.

The geometric model (Fig. 1a) consists of a circular bundle of WM fibers surrounded by a reference medium. The WM fiber bundle is divided into 3 types of micro-compartments corresponding to axon, myelin and extra-axonal space. For ease of computation, the model is calculated in 2D as a single plane transecting the WM fiber bundle. Each axon is modeled as an infinite cylinder with myelin represented as an annular ring surrounding the axon with a pre-defined g-ratio (the ratio of inner to outer diameter of the myelin sheath Rushton, 1951) of 0.65 ± 0.1 (Albert et al., 2007; Jacobs and Love, 1985) unless otherwise stated. Axons are densely-packed at random location using a circle-packing algorithm (Collins and Stephenson, 2003). The volume fraction of WM fibers used was approximately 0.7 (assuming 0.2 extracellular space Nicholson and Syková, 1998; Syková, 2005 and 0.1 glia volume fraction Lehre and Rusakov, 2002). The WM fibers have a mean diameter of approximately 1 μm and the fiber diameter follows a gamma distribution (Aboitiz et al., 1992). The WM fibers are approximately parallel with slightly randomized orientation (angular variance of 5°). The magnetic susceptibility of the myelin compartment is − 0.08 ± 0.01 ppm (Lee et al., 2012; Liu et al., 2011) and the axonal and extra-axonal compartments and reference space are set at 0 ppm (unless otherwise specified). *T*_2_ values for the myelin and extra-axonal compartments were set to 25 ms and 75 ms, respectively (Laule et al., 2004). The axonal bundle is simulated for a range of orientations (*θ*) with respect to *B*_0_ (by rotating *B*_0_ relative to the simulated 2D plane). For realism, the magnetic susceptibility, g-ratio and WM fiber orientations are randomly varied from one axon to the next. The magnetic field perturbation (see Fig. 1b) was calculated using analytical solutions (Haacke et al., 1999). Magnetization evolution at each grid point is calculated using Bloch equations with short time steps, and the signal is calculated as the complex sum over the WM fiber bundle (excluding the reference medium, Fig. 1d). The proton density in the myelin compartment was assumed to be half that of the other compartments. The 2D model was simulated over a FOV of 0.35 mm × 0.35 mm to calculate signal for an area roughly comparable to an MRI pixel. A high resolution grid space was used to ensure that the magnetic field perturbation pattern for each axon is sufficiently represented. After extensive testing of the trade-off between accuracy and computational feasibility, the grid was chosen such that 28 × 28 grid points covers 1 × 1 μm^2^ (exactly framing an axon of median diameter) and the simulated FOV comprises approximately 7000 × 7000 grid points with 50,000 axons.

In this geometric model, we utilized the analytical solution for the magnetic field perturbation caused by an infinite cylinder (Haacke et al., 1999) to calculate the magnetic field change caused by each WM fiber at a given orientation to *B*_0_. The analytical solution for the magnetic field perturbation at a given grid point due to one axon is given by Eqs. (1)–(3), where subscripts indicate compartments (*ax* = axonal, *ea* = extra-axonal, *my* = myelin), Δ*f* is the change in resonance frequency in *Hz*, Δ*χ* is the difference in magnetic susceptibility with respect to *χ*_ea_, *θ* is the orientation of the cylinder's long axis to *B*_0_, *R* is the radius of the compartment, *ϕ* and xtitr are the polar coordinates at each grid point with reference to the direction of *B*_0_ and the centre of the axon. For a given grid point and axon, we select between Eqs. (1) and (3) depending on which compartment the grid point lies in with respect to that axon (i.e., Δ*f*_ea_ is used for all grid points external to that particular axon, even if the grid point lies inside another WM fiber). WM fiber orientations were varied by changing *θ*, which is an approximate and simplistic method for adding some angular variation to an otherwise perfectly parallel fiber system. Thus, although the axons are simulated as perfect circles in the simulation, the field effects reflect a range of orientations. This calculation is summed over all axons. Simulation was performed using Matlab (R2010a, Mathworks, Natick, MA) requires a total processing time for one simulated FOV of about 1 hour when parallelized on a 100-node cluster.

(1) $$\Delta f_{\mathrm{ax}}=\frac{1}{2}B_{0}\gamma \Delta \chi_{\mathrm{ax}}\left[\cos^{2}\theta -\frac{1}{3}\right]$$

(2) $$\Delta f_{\mathrm{my}}=\frac{1}{2}B_{0}\gamma \left\{\Delta \chi_{\mathrm{my}}\left[\cos^{2}\theta -\frac{1}{3}\right]-\left[\Delta \chi_{\mathrm{my}}-\Delta \chi_{\mathrm{ax}}\right]{\left(\frac{R_{\mathrm{ax}}}{r}\right)}^{2}\sin^{2}\theta \cos\left(2\phi \right)\right\}$$

(3) $$\Delta f_{\mathrm{ea}}=\frac{1}{2}B_{0}\gamma \sin^{2}\theta \cos\left(2\phi \right)\left\{\Delta \chi_{\mathrm{my}}{\left(\frac{R_{\mathrm{my}}}{r}\right)}^{2}-\left[\Delta \chi_{\mathrm{my}}-\Delta \chi_{\mathrm{ax}}\right]{\left(\frac{R_{\mathrm{ax}}}{r}\right)}^{2}\right\}$$

### MRI acquisition and data processing

In-vivo experiments on seven healthy volunteers were conducted on a 3.0 T Siemens Trio MRI scanner with a 12-channel head coil. A 2D multi-echo GRE pulse sequence was used to obtain phase and magnitude images at 128 echo times (FOV 96 × 96 mm^2^; resolution 2 × 2 × 2 mm^3^; 5 axial slices with 12 mm separation; flip angle 10°; TR 1.5 s; TE 4-260 ms; ∆TE 4 ms; scan time 25 min). The *R*_2_* value was estimated with least-squares linear fitting to the logarithm of the magnitude images for echoes from 4 to 148 ms. Unwrapping of the signal phase was performed on a voxel-by-voxel basis in 1D (Matlab) on echoes acquired from 4 to 60 ms, and fed into least-squares linear fitting to determine voxel mean frequency. Background field variation was removed by subtracting a low pass filtered phase image from the original phase image (5 × 5 mean filter). DTI data (2 × 2 × 2 mm, b = 1000 *s*/*mm*^2^, 30 directions) was acquired in the same session and used to determine the orientation dependence of WM fibers relative to *B*_0_, and to create a WM mask (FA > 0.3). Eleven bins were used to classify the orientation of the WM fibers with bin-width proportional to sin*θ* (thus avoiding a paucity of voxels contributing at the lowest angles). To characterize the signal evolution over time, the deviation from linear phase and mono-exponential magnitude decay were calculated by subtracting off the fitted linear phase and *R*_2_* decay from the phase and magnitude time courses, respectively. This was calculated separately for the average time courses corresponding to approximately parallel (0°–15°) and perpendicular (75°–90°) WM fibers.

**Fig. 2:**
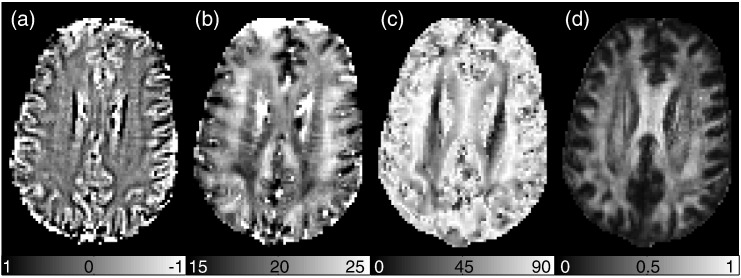
In-vivo dependence of phase and *R*_2_* on orientation of WM fibers to ***B*_0_**. (a) Phase map. (b) *R*_2_* map. (c) Map of angle between principle eigenvector with ***B*_0_** from DTI. (d) Fractional anisotropy (FA).

We compared several model predictions to GRE signal measured in seven volunteers at 3 T. In the first instance, we considered the orientation dependence of GRE phase and *R*_2_* which has recently been reported by several groups (Bender and Klose, 2010; Cherubini et al., 2009; Denk et al., 2011; He and Yablonskiy, 2009; Lee et al., 2010a, 2011; Liu, 2010; Marques et al., 2009; Sati et al., 2012). The predominant angle of WM fibers to *B*_0_ was extracted from diffusion tensor imaging (DTI) data acquired in the same scan session (see Fig. 2). We also studied the time evolution of the signal phase and magnitude, which has received less attention (van Gelderen et al., 2012). Model predictions for the temporal deviation from linear phase and mono-exponential decay were compared to measurements.

## Results

### Phase modulation with orientation

**Fig. 3:**
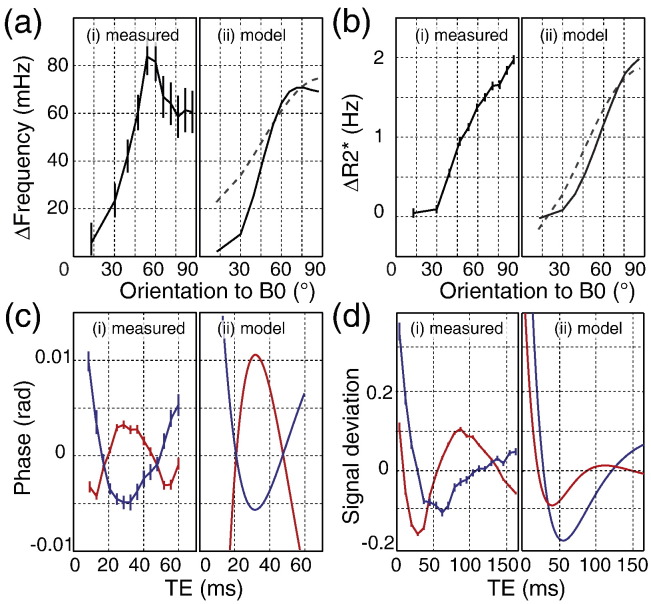
Frequency and *R*_2_* modulation with orientation to ***B*_0_**. (a) Measured and simulated frequency modulation. (b) Experimental and simulated *R*_2_* modulation. For (a) and (b) the dashed line represents the best fit sin^2^*θ* dependence proposed previously. (c) Experimental and simulated deviation from linear phase evolution (signal phase minus linear fit). (d) Experimental and simulated deviation from mono-exponential decay (signal magnitude minus *R*_2_* decay fit). For (c) and (d) the blue and red lines represent parallel and perpendicular WM fibers respectively. Error bars indicate the standard error across all subjects.

Fig. 3a shows the measured and simulated dependence of the signal phase (reflecting the mean of the frequency distribution) on orientation to *B*_0_. The measured phase of WM fibers approximately perpendicular to *B*_0_ (*θ* ≈ 90°) is positive relative to WM fibers approximately parallel to *B*_0_ (*θ* ≈ 15°), in agreement with previous studies (Cherubini et al., 2009; Denk et al., 2011; Lee et al., 2010a). The frequency (converted directly from the phase) increases with *θ* up to a peak between 45°–65°, and then decreases after the peak (Fig. 3ai). A similar trend^1^ was reported previously by Denk et al., 2011. The orientation dependence of phase predicted from our model (black line in Fig. 3aii) exhibits a similar peak, albeit less pronounced and at slightly higher angle 65°–75°. Most strikingly, this forward model (using parameters taken from literature) predicts a comparable range of frequencies as the measured data.

Two models have been proposed for orientation dependence of GRE signal phase: the generalized Lorentz cavity (He and Yablonskiy, 2009) and susceptibility anisotropy (Lee et al., 2010a). The generalized-Lorentz model predicts a sin^2^*θ* dependence induced by the presence of cylindrical, susceptibility-shifted inclusions. In fitting this model to their data, Denk et al., 2011 required ad hoc scaling of the frequency axis to account for the observed phase peak (Denk et al., 2011). The susceptibility anisotropy model assumes an orientation dependence of the magnetic susceptibility itself, which can occur in certain biological structures like membranes, and also follows a *sin*^2^*θ* dependence. Fitting a *sin*^2^*θ* dependence to the experimental data produces a poor fit (*R*^2^ = 0.584, dashed line in Fig. 3aii). Both the modified-Lorentz and susceptibility-anisotropy models calculate the phase orientation dependence for a single compartment, which may in general contain fractional contributions from multiple species with varying susceptibility. Our model builds on these ideas by explicitly incorporating the micro-geometry of the WM compartments to establish the contribution of microstructural compartmentalization, and provides a more accurate description of this effect.

### *R*_2_* modulation with orientation

Fig. 3b shows the measured and simulated dependence of *R*_2_* (reflecting the frequency distribution variance) on orientation to *B*_0_. The experimental *R*_2_* results exhibit a roughly sinusoidal increase with *θ* (Fig. 3bi) spanning about 2 Hz, in good agreement with previous reports (Bender and Klose, 2010; Denk et al., 2011). The model simulation matches quite well with the experimental results (black line in Fig. 3bii). In the limit of low volume fraction, parallel cylinders of susceptibility-shifted material are predicted to have a *sin*^2^*θ* dependence (Yablonskiy and Haacke, 1994), which produces a good fit to the data (adjusted *R*^2^ = 0.976 dashed line in Fig. 3bii). However, at the smaller angles (< 30°) the change in R_2_* with respect to angle as predicted by the *sin*^2^*θ* trend is larger than our experimental data. The *sin*^2^*θ* trend typically considers only the extra-cylinder component and at 0°, no signal decay due to magnetic field inhomogeneity would be expected. In our simulation, even at 0° the presence of susceptibility shifted frequencies cause an interference pattern that drives signal decay. Previous work on fixed brain at 7 T has reported a *sin*^4^(*θ*) dependence, which was attributed to myelin magnetic anisotropy (Lee et al., 2011). Fitting a *sin*^4^(*θ*) dependence corresponds to an adjusted *R*^2^ value of 0.977. A p-value of 0.792 was obtained from a F-test comparing the fit with and without an additional *sin*^4^(*θ*) dependence suggesting that our data at 3 T does not display a significant *sin*^4^(*θ*) dependence.

The simulated baseline *R*_2_* value at *θ* ≈ 15° is smaller than what was measured from the experiments (16 Hz from our simulation vs 18.5 Hz measured from our experiments), which likely reflects additional sources of signal decay which were not considered in our simulations (e.g., imperfect shim). In addition, the contributions from other substructures such as iron-rich oligodendrocytes and hemoglobin-bearing blood vessels were not explicitly modeled in our model. The inclusion of these structures would contribute further field perturbations, thereby shifting the baseline of *R*_2_* but is not expected to introduce orientation dependence. The approximately spherical shapes of the oligodendrocytes (Pannese, 1994) suggest that their contribution would generate orientation-invariant magnetic field perturbations (Haacke et al., 1999). WM vasculature is generally aligned with the WM fibers, but has been reported to have insignificant contribution to phase contrast due to the low volume fraction (even in gray matter) (Lee et al., 2010b; Marques et al., 2009; Weber et al., 2008).

### Signal phase evolution

We investigated how the signal phase evolution deviates from linear phase accrual (Schweser et al., 2011; Wharton and Bowtell, 2012). Analysis of the experimental phase evolution against time revealed significant deviation of the measured phase from a linear relationship with echo time. The phase residuals (after subtraction of the linear fit) are approximately quadratic forms, with negative and positive coefficients for perpendicular and parallel WM fibers respectively (Fig. 3ci). The geometric model predicts very similar trends with echo time, and replicates the difference between parallel and perpendicular WM fibers (Fig. 3cii). Linear phase evolution implies that there exists a central frequency about which signal from positive and negative frequency offsets cancels at all timepoints. Deviations from this behavior indicate this cancellation does not happen, either because the frequency distribution is not symmetric or because *R*_2_ signal decay correlates to frequency, either of which implies compartmentalization. Compartmentalization of *R*_2_ (e.g., in myelin) will influence the shape of the signal phase evolution; however, the dependence on orientation indicates that susceptibility also plays a role in this effect (since *R*_2_ is orientation independent Henkelman et al., 1994). This phenomenon illustrates an important difference between our model and the simpler approach of approximating the distribution from the field generated outside of a single cylinder characterized by a mean magnetic susceptibility: the latter model always predicts a linear phase evolution, while ours encapsulates non-linear evolution.

### Signal magnitude evolution

The residual time course of the signal magnitude (subtracting the *R*_2_* fit) revealed significant deviation from mono-exponential decay (Fig. 3di), with different characteristic shapes for parallel and perpendicular WM fibers. Simulated results predicted similar trends (Fig. 3dii). Non-mono-exponential behavior of GRE signal decay in WM has been previously reported (Du et al., 2007; van Gelderen et al., 2012). An early study attributed this behavior to the presence of a myelin water pool with a short *T*_2_ and a larger water pool with longer *T*_2_ corresponding to intra- and extra-axonal water (MacKay et al., 1994). More recently, it has been suggested that these water pools are also phase shifted with respect to each other (van Gelderen et al., 2012). In our experiments, parallel and perpendicular WM fibers exhibit distinct oscillatory behaviors that are consistent with the presence of compartments with magnetic susceptibility differences. With respect to signal inflection points, the deviation curve for parallel WM fibers are expanded in time relative to the perpendicular WM fibers, which may reflect a narrower frequency distribution. Signal inflection points have also been shown previously to occur at shorter echo times as field strength increases, consistent with a susceptibility-driven effect (van Gelderen et al., 2012). The simulated signal decay from each individual compartment exhibits Gaussian-like decay at the early time points (Yablonskiy and Haacke, 1994), which contributes to the deviation from mono-exponential decay, but is not the primary factor driving the shape of these curves (as indicated by a poor fit to a Gaussian). Deviations from mono-exponential decay were not due to low-spatial-frequency background gradients caused by poor shim and macroscopic susceptibility differences interfaces, as confirmed by both a fieldmap simulation of gradient-induced signal dephasing and explicit calculation using the model described by Yablonskiy and Haacke (1994).

### Susceptibility anisotropy

**Fig. 4:**
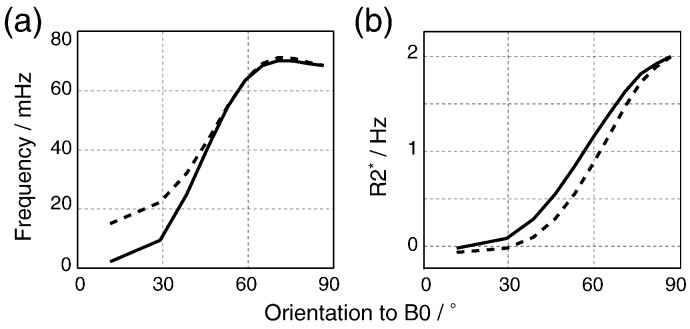
Effects of susceptibility anisotropy on orientation modulation. (a) Simulated frequency modulation. (b) Simulated *R*_2_* modulation. Solid line represents simulation without susceptibility anisotropy while dashed line represents simulation with susceptibility anisotropy.

Previous work has considered susceptibility anisotropy as a bulk property to explain orientation dependence (Lee et al., 2010a; Lee et al., 2012; Liu et al., 2011). Within our geometric model, this effect would operate at a compartmental level, most likely in the myelin (Lee et al., 2010a). We incorporated susceptibility anisotropy in the myelin compartment. Here, we assume the most basic form of susceptibility anisotropy which corresponds to a cylindrically symmetric susceptibility tensor with principal axis along the long fiber axis, which can be written as *χ*_my_(*θ*) = *χ*_iso_ + *χ*_aniso_ × sin^2^(*θ*) (Lee et al., 2010a), with *χ*_aniso_ = − 0.04 ppm (which is the susceptibility anisotropy measured on lecithin membranes using phase contrast microscopy Boroske and Helfrich, 1978) and *χ*_my_(90°) = − 0.08 ppm. Susceptibility anisotropy results in a phase modulation with a slightly reduced range of values, but the general trend remains, including the frequency peak between 65° and 75° (Fig. 4a). The resultant *R*_2_* modulation is again similar, with susceptibility anisotropy altering the specific shape but preserving the overall trends (Fig. 4b). These simulations suggest that, while susceptibility anisotropy is not inconsistent with our results, neither is it required to generate the general effects seen in the experimental data.

## Discussion

### Geometric model

#### Frequency map formulation

In general, to find the magnetic field perturbation caused by any object or ensemble of objects, iterative numerical methods are required to solve the underlying partial differential equation boundary value problem which are computationally expensive. However, algebraic solutions are readily available for regular ellipsoidal objects and the magnetic field perturbation at any point in space caused by an ensemble of ellipsoidal objects can be calculated as the summation of the field perturbation caused by each individual ellipsoidal object (Schenck et al., 1996). This approach has been widely utilized in modeling a range of biological systems such as blood vessel network, bone marrow and the trabecular network (Yablonskiy and Haacke, 1994). In particular, infinite cylinders are frequently used to model susceptibility-shifted structures such as blood vessels and the related BOLD signal (Boxerman et al., 1995; Ogawa and Lee, 1990; Ogawa et al., 1993). The highly asymmetric aspect ratio of axons (with a small diameter compared to the length of a straight segment) should make infinite cylinders even better proxies of WM fibers than blood vessels. The closed form solution to the magnetic field perturbation for a single infinite cylinder forms the building block of our model (Haacke et al., 1999). As shown below, it is straightforward to modify this calculation to describe nested cylinders representing axons covered by a myelin sheath. At each grid point, the frequency is given by the summation of field offsets contributed by each axon, resulting in a highly-accurate (albeit inefficient) calculation.

A fast and efficient method for calculating the magnetic field perturbation from arbitrary shapes has been recently introduced (Marques and Bowtell, 2005; Salomir et al., 2003). By treating the field perturbation map as a convolution of the underlying susceptibility distribution with a unit dipolar field, an element wise multiplication in the Fourier domain can be performed. This Fourier method allows the calculation of magnetic field perturbation from objects of arbitrary shapes and has been shown to agree well with the closed-form analytical solution for regular ellipsoids. Our initial implementation used Fourier calculations to enable fast calculation of more complex geometries. However, the Fourier method induces significant errors at the compartment boundaries, which can only be avoided with increased grid resolution, reducing the computational gains. The abundance of compartment boundaries in our model were observed to accumulate significant errors in signal behavior. The Fourier method further suffers from Gibbs ringing due to the finite extent of the real and k-space space domains, requiring a FOV at least double the size of the simulated object, quadrupling the number of points in the 2D grid. For these reasons, the Fourier approach was abandoned in favor of the spatial calculations discussed above.

The Fourier approach above was initially favored as it enabled a 3D model, in which the axons were modeled as elliptical (rather than circular) cylinders and spheres were included to represent oligodendrocytes. A 3D model would further allow more sophisticated geometries to be described, such as non-parallel fibers (e.g. crossing fibers). However, the computational overheads of 3D modeling ultimately limit the size of the FOV that can be reasonably modeled. The result is a less stable signal calculation that includes fewer axons and is sensitive to the specific geometry that is simulated. We conducted tests to consider the benefit of including more realistic, ellipsoidal cylinders (or even less regular geometries) and concluded that this had a relatively minor effect on the simulated signal. The difficulty simulating oligodendrocytes likely represents a greater compromise to the model, which may affect the signal decay time, but not orientation, dependence. This will be considered in future model improvements.

#### Existing geometric models

There is a long-standing literature on modeling the MRI signal from microscopically heterogeneous tissue. One approach models tissue using susceptibility shifted objects (“perturbers”) embedded in a homogeneous medium (Yablonskiy and Haacke, 1994). Perturbers are modeled using regular shapes such as cylinders and spheres, and one can then calculate the resultant magnetic field and the subsequent signal phase and/or magnitude evolution. This is essentially a single compartment model because signal change is only considered in the medium (not the perturbers) and the field offsets induced by the perturbers are assumed not to overlap, both of which are reasonable assumptions if the volume fraction of the perturbers is low. These models have been very successful in characterizing systems such as trabecular bone (Majumdar, 1991), blood vessel networks (Boxerman et al., 1995; Ogawa et al., 1993) and contrast agents (Boada and Haacke, 1993). The extension of a similar approach to study MR signal behavior in WM is non-trivial due to the presence of signal from multiple compartments and the significant volume fraction of each of these compartments. The interaction of signal from the different compartments causes the deviation of the signal behavior from that predicted by a single compartment model.

Wharton and Bowtell (2012) has previously introduced a similar hollow-cylinder model by looking at the field perturbation caused by a representative single white matter fiber incorporating isotropic and anisotropic magnetic susceptibility and chemical exchange. By fitting compartmental *T*_2_, g-ratio, exchange rates etc, that work reproduced experimentally observed non-linear phase evolution trends in WM. A major difference in our model is that we explicitly model the microstructural arrangement of the densely packed WM fibers (e.g packing, distribution, diameters, etc).

#### Packing geometry

**Fig. 5:**
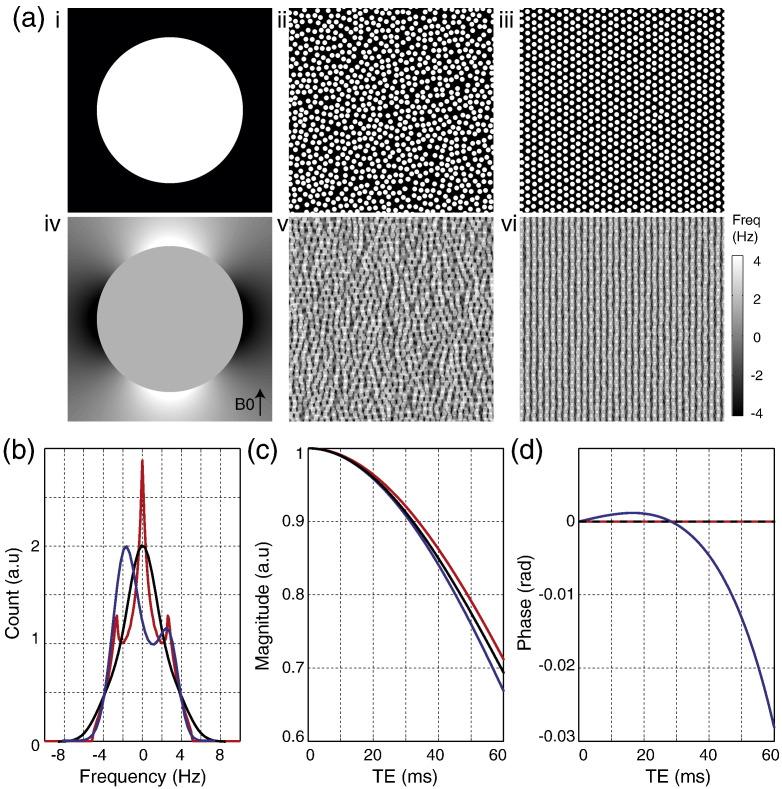
Effects of cylinder geometry on magnetic field perturbation. (ai) represents a single cylinder. (aii) represents a random distribution of smaller cylinders. (aiii) represents a hexagonal packing of smaller cylinders. (ai)–(aiii) have the same volume fraction of 0.4 and magnetic susceptibility of -0.08 ppm. The field maps generated by the single cylinder is shown in (aiv), random packed smaller cylinders (av) and hexagonal packed smaller cylinders (avi). (b) shows the frequency histograms of the extra-cylinder compartment from (aiv) to (avi). (c) shows the corresponding magnitude decay. (d) shows the signal phase evolution. Red, black and blue represent the single cylinder, random packed cylinders and hexagonal packed cylinders respectively.

One of the crucial realizations in developing this model is the importance of packing geometry due to the high volume fraction of axons. In the low-volume regime, one can accurately approximate the frequency distribution for a set of perturbers from the field pattern surrounding a single perturber (cylinder or sphere) (Yablonskiy and Haacke, 1994). However, once the perturbers have a high volume fraction, the fields surrounding adjacent perturbers interact and this approximation becomes inaccurate. To illustrate this, consider a single cylinder with volume fraction of 0.4 and susceptibility of − 0.08 ppm (Fig. 5) and a random packing of smaller cylinders with the same volume fraction. The frequency histograms of the single cylinder and the multiple smaller cylinders are markedly different. The histogram of the single cylinder shows 3 distinct peaks with the 2 side peaks contributed by the dipolar field pattern of the cylinder. On the other hand, the random packing of smaller cylinders smooth out the distinct dipolar pattern from each individual cylinder and resulted in a single peak frequency histogram. The decay rate of the single cylinder can also be seen to differ from that of a random distribution of smaller cylinders with the same volume fraction. The distribution of cylinders within an ensemble of cylinders also affects the signal behavior. Here, we consider the field perturbation caused by hexagonally packed cylinders (volume fraction = 0.4). The frequency histogram of the hexagonally packed cylinders is distinctly different from the randomly packed cylinders, the histogram of the hexagonal pack of cylinder is asymmetric with 2 distinct peaks. As expected, the signal decay is different from the randomly packed cylinders. An interesting observation is the non-linear phase evolution shown by the hexagonally packed cylinders which is caused by the asymmetry in the frequency distribution in the absence of compartmentalization of different *T*_2_ since we only considered the extra-cylindrical compartment. Note that for both random and hexagonal cylinder packing, the bulk shape of the packing is a larger circle and only a square roi at the middle of the circular packing was considered. *B*_0_ direction is the same for all scenarios.

### Effect of tissue parameters

Additional simulations were conducted to consider the contribution of several key tissue parameters.

#### Magnetic susceptibility

**Fig. 6:**
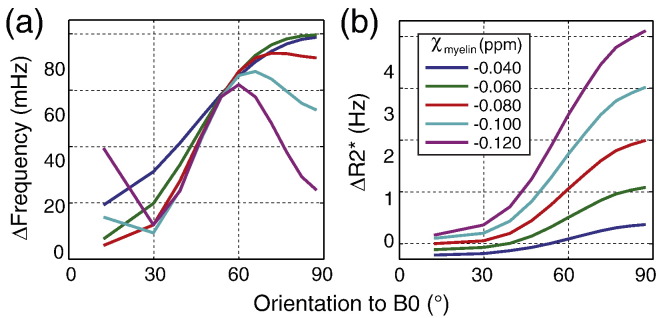
Effects of different magnetic susceptibility values. The magnetic susceptibility of myelin compartment was varied from − 0.12 ppm to − 0.04 ppm and that of the axonal and extra-axonal compartment was kept at 0 ppm. (a) Orientation modulation of frequency. (b) Orientation modulation of *R*_2_*.

In general, the signal magnitude and phase are dependent on the magnetic susceptibilities of the various compartments. As an illustration, We showed the effect of varying magnetic susceptibility values for the myelin compartment on the orientation dependence to *B*_0_. The magnetic susceptibility of myelin was varied from − 0.12 ppm to − 0.04 ppm while keeping the magnetic susceptibility of the axonal and extra-axonal compartments at 0 ppm. TEs and all other modeling parameters are the same as in methods. As shown in Fig. 6, changing the magnetic susceptibility of myelin affects both frequency and decay modulation. For frequency modulation, the general trend of the modulation curve changes significantly with different susceptibility values. When the susceptibility difference is small (e.g. |*χ*_my_| = 0.04 ppm), an approximately linearly increasing trend was observed. When the susceptibility difference is further increased to 0.06 ppm, a more sine-like increase was observed. At 0.08 ppm, the peak of the modulation curve shifts to an angle less than 90°. The peak between 60° to 90° continues to occur at a smaller angle as the susceptibility difference increases. Another interesting observation is the decrease infrequency change from 0° to 30° when the susceptibility difference at larger than 0.1 ppm. For signal decay modulation, an increase in the magnetic susceptibility susceptibility difference of myelin causes an increase in the decay rates, with an approximate increase of 1 Hz for every 0.02 ppm increase in |*χ*_my_| (within the simulated range of *χ*_my_). An increase in magnetic susceptibility of myelin results in a larger magnetic field perturbation pattern with a wider range of frequencies present and thus increases the decay rate.

#### Diffusion

**Fig. 7:**
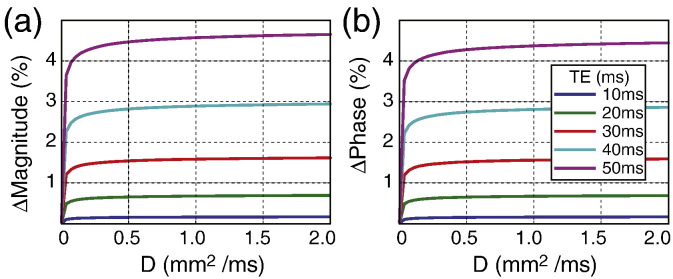
Effects of diffusion. The percentage change in signal magnitude (a) and signal phase (b) at different simulated diffusion coefficient for different TEs.

The static dephasing regime was assumed for the simulation results shown in the paper. To investigate the effect of diffusion on the simulation results, Monte Carlo simulation was performed on 50,000 spins. Spins were randomly placed in the 2D frequency map corresponding to a fiber orientation of 90° (which results in the largest field perturbations and therefore greatest sensitivity to diffusion). Each spin undergoes a random walk with compartment-specific diffusion coefficient. The diffusion coefficient of spins in the axonal and extra-axonal compartments was varied from 0 to 2 μm^2^ ms^− 1^. A time step of 0.5 μs and up to 100,000 time steps were used. Spins present in the myelin are assumed to be stationary (see below). All boundaries are impermeable and spins are reflected at the boundaries (by treating the boundaries as being perpendicular to the 2D plane). The effects of varying diffusion coefficient on the signal magnitude and phase at different echo times (from 10 ms to 50 ms) was investigated. Simulation results (Fig. 7) show an initial change in signal magnitude and phase, which then taper off asymptotically. These simulations suggest that the change in the signal due to diffusion is relatively small and should not significantly affect the simulations calculated without diffusion effects (i.e., the static dephasing regime is a sensible approximation). In addition, the simulations demonstrate that the dependence on diffusion coefficients is negligible for the range of values found in white matter (0.1–1 μm^2^ ms^− 1^), indicating that the orientation dependence should not be driven by preferential diffusion along axons. The assumption of negligible diffusivity in myelin is important to this result, as the myelin sheath is the region of greatest field heterogeneity. MRI studies have demonstrated that diffusion within myelin is lower than intra- or extra-cellular diffusion by at least a factor of two in peripheral nerves (Andrews et al., 2006), while a recent microscopy study of myelin in functional axons concluded that diffusion is “profoundly slower” in axons in the central nervous system compared to peripheral nerves (Velumian et al., 2011). These studies would suggest that negligible diffusion within myelin is a reasonable assumption.

#### *T*_2_ of myelin compartment

**Fig. 8:**
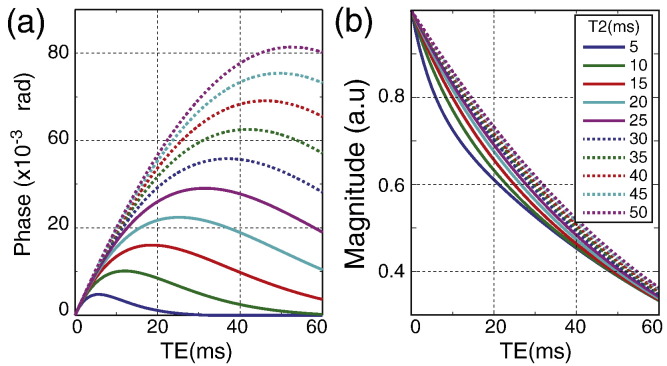
Effects of varying myelin *T*_2_ (a) shows the change in signal phase over time for a range of simulated *T*_2_ of myelin. Panel (b) shows the change in signal magnitude over time for a range of simulated *T*_2_ of myelin.

The susceptibility-shifted myelin compartment plays an important role in modulating the signal magnitude and phase behavior. However, the non-mono-exponential decay and non-linear phase accrual will also be affected by the fast decay of myelin signal (Du et al., 2007). As such it is expected that changing the *T*_2_ of the myelin compartment would also change behavior of both the signal phase and magnitude decay. From Fig. 8, it could be seen that increasing *T*_2_ of myelin shifts the peak of the phase curve to the right and produces a larger phase change. The log(magnitude) curve becomes more linear with increasing *T*_2_ indicating that the signal magnitude decay become more mono-exponential within a TE range of 0 to 60 ms. These results indicate that both the *T*_2_ and magnetic susceptibility of myelin modulates the signal temporal dynamics. However, *T*_2_ cannot create the profound orientation dependence observed in the temporal dynamics of both phase and magnitude signal, indicating that susceptibility plays a crucial role in the observed temporal dynamics.

### TE dependence of R_2_* and mean frequency offset

The GRE signal deviation from monoexponential decay and linear phase evolution have direct implications on *R*_2_* and GRE phase imaging: the calculated *R*_2_* decay rates and mean phase offsets are dependent on the range of TEs chosen. In this study, we wanted to maximize the number of echoes used in the both the fitting of R2* and frequency mapping in order to display a wider range of non-monoexponential decay and non-linear phase evolution characteristics. However, the number of echoes used in each fitting is also limited by the signal to noise ratio (SNR) as SNR decreases as TE increases. We performed a simple visual inspection on the magnitude and phase images and concluded that the SNR for magnitude images was sufficient up to 148 ms and that for phase images was up to 60 ms. As such, TEs of up to 148 ms were used to investigate in the *R*_2_* fitting and TEs of up to 60 ms were used in the frequency fitting.

## Conclusions

We have presented a 2D geometric model that represents the shapes and orientations of WM substructures, their arrangement with respect to one another and their magnetic susceptibility. Crucially, this forward model uses parameter values based on literature rather than fitting the model to the experimental results. The success of this simple model in predicting a broad range of signal properties provides confidence that signal behavior is, in fact, driven by the modeled microstructure. The power of this approach is in generating the entire frequency profile, rather than simply the low-order moments, leading to a much richer description of signal in the presence of microstructural changes. This approach may be used to systematically predict signal behavior in disease, in the hope of identifying characteristic biomarkers. For example, one might hope to separate the effects of decreasing myelin thickness and increasing iron concentration in multiple sclerosis. Given the presence of multiple susceptibility species in WM, robust characterization of microstructure may require use of the full richness of information contained in the intra-voxel frequency distribution, rather than the low-order moments reflected in phase and *R*_2_* mapping.

## Footnotes

1 Note that there is ambiguity in the sign of phase measurements, and the scanner used by Denk et al., 2011 adopts the opposite convention to ours.
